# Improving Outcomes of Liver Transplantation for Polycystic Disease in MELD Era

**Published:** 2013-02-01

**Authors:** R. F. Saidi, N. Jabbour, S. A. Shah, Y. Li, A. Bozorgzadeh

**Affiliations:** *Division of Organ Transplantation, Department of Surgery, University of Massachusetts Medical School, Worcester, MA, USA*

**Keywords:** Liver transplantation, Polycystic liver disease, MELD, Transplantation outcome

## Abstract

Background: Liver transplantation (LT) for polycystic liver disease (PLD) has evolved to be an option for treating these patients. Patients with PLD suffer from incapacitating symptoms because of very large liver volumes but liver function is preserved until a late stage.

Objective/Methods: Herein, we reviewed the outcome of adult patients with PLD who underwent LT in the US comparing pre-MELD (1990–2001) to MELD era (2002–2009).

Results: During this period, only 309 patients underwent LT for PLD. The number of LT for PLD is very low comparing the two eras. The percentage of patients who had combined liver and kidney transplantation (CLKT) for this disease has not changed during MELD era (42.8% *vs* 38.6%). The waiting time for LT (337 *vs* 272 days) and CLKT (289 *vs* 220) has increased in MELD era (p<0.001). In MELD era, 53.4% of LT and 31.2% of CLKT were done as MELD exceptional cases. The allograft and patent survival have significantly improved in MELD era.

Conclusion: Patients with PLD had marked improvement of their outcomes after LT in MELD era.

## INTROCUCTION

LPolycystic liver disease (PLD) is a rare, hereditary, benign disorder. Hepatic failure is uncommon and symptoms are caused by mass effects leading to abdominal distension and pain. PLD has an autosomal dominant inheritance and is characterized by the presence of multiple scattered cysts of biliary origin in the liver parenchyma [1-3]. Liver transplantation (LT) is the only curative option [3-6]. We conducted this study to compare the outcome of LT in patients with PLD before and after MELD.

## MATERIAL AND METHODS

We reviewed the files of patients with PLD who underwent LT during 1990–2009, as reported in the UNOS database, and compared their outcome pre-MELD (1990–2001) with MELD era (2002–2009).

## RESULTS

**Figure 1:**
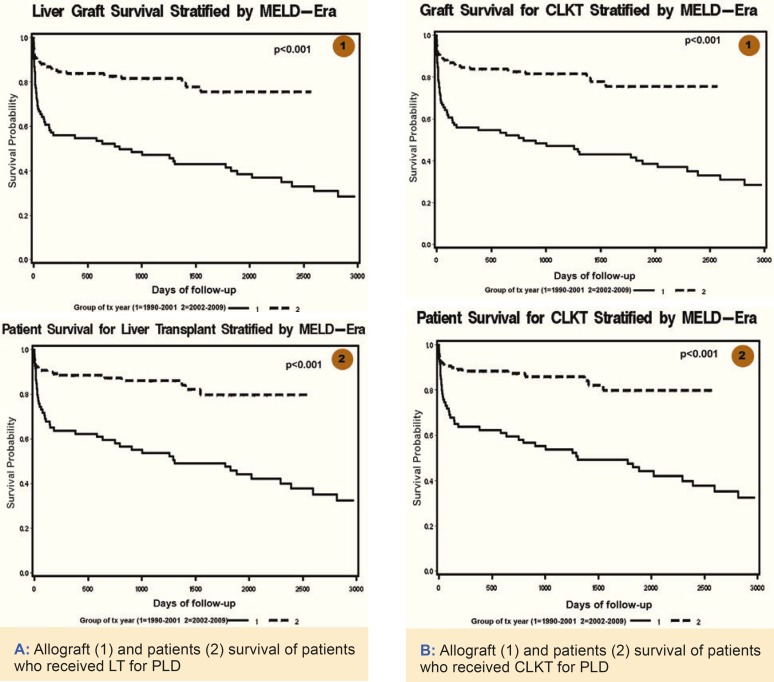
Allograft and patients survival after LT (A) or CLKT (B) for PLD. In MELD (dashed line) *vs* pre-MELD era (solid line).

During this period, only 309 patients underwent LT for PLD. The number of LT for PLD is very low comparing the two eras. The percentage of patients who had combined liver and kidney transplantation (CLKT) for PLD has not changed during MELD era (42.8% *vs* 38.6%). The waiting time for LT (337 *vs* 272 days) and CLKT (289 *vs* 220) has increased in MELD era (p<0.001). In MELD era, 53.4% of LT and 31.2% of CLKT are done as MELD exceptional cases (Table 1). The allograft and patent survival have significantly improved in MELD era (Fig 1).

**Table 1:** Patients characteristic

Variables	pre-MELD era	MELD era	P
	150/40730 (0.4%)	159/42601 (0.4%)	
Age	48.5	51.9	NS
MELD	17.1	17.9	NS
Length of stay (LOS)	22.9	17.5	<0.001
Wait time	272	337	<0.001
CLKT	58 (38.6%)	68 (42.8%)	
on dialysis	28.4%	47.1%	0.02
MELD	24.5±7.5	25±6.3	NS
LOS	25	19	NS
Wait time	220±274	289±331	<0.001

## DISCUSSION

PLD is a rare disease with a prevalence of 0.05% to 0.13% in autopsy series. It is considered a benign disease, but its progression can result in substantial morbidity, and this in turn can increase mortality rates [1-3]. LT has been utilized in these patients with acceptable results in small case series [3-6].

During the MELD era, the outcome of LT for PLD improved dramatically. However, the waiting time also increased. Interestingly, the number of patients who underwent CLKT did not change in MELD era. It seems that PLD patients had a disadvantage in MELD era.

This is a retrospective analysis of the UNOS data. We recognize both potential advantages and limitations of this study that used a large national database. However, the larger sample size provides sufficient power to detect independent risk factors that may usually be missed in single-center studies. As with any analysis utilizing the UNOS database, our conclusions rely on the assumption that there is no systematic bias generated by reporting error or missing data. However, the primary endpoint for this analysis was allograft and patient survival, which is reliably captured in the UNOS database. Residual or unmeasured confounders that could impact allograft and patient survival including differences in immunosuppression protocols, the fat content/quality of the allograft and center-specific practices were not available in the database.

In conclusion, patients with PLD had marked improvement of their outcomes after LT in MELD era.

## References

[1] van Keimpema L, de Koning DB, van HB (2011). Patients with isolated polycystic liver disease referred to liver centers: clinical characterization of 137 cases. Liver Int.

[2] Everson GT, Taylor MR, Doctor RB (2004). Polycystic disease of the liver. Hepatology.

[3] Pirenne J, Aerts R, Yoong K (2001). Liver transplantation for polycystic liver disease. Liver Transpl.

[4] Ueno T, Barri YM, Netto GJ (2006). Liver and kidney transplantation for polycystic liver and kidney-renal function and outcome. Transplantation.

[5] Krohn PS, Hillingso JG, Kirkegaard P (2008). Liver transplantation in polycystic liver disease: A relevant treatment modality for adults?. Scand J Gastroenterol.

[6] Kirchner GI, Rifai K, Cantz T (2006). Outcome and quality of life in patients with polycystic liver disease after liver or combined liver-kidney transplantation. Liver Transpl.

